# Tropical Dysentery

**Published:** 1860-11

**Authors:** Richard Whittingham

**Affiliations:** Surgeon Peruvian Navy


*On Tropical Dysentery.* By Richard Whittingham, M. D., Surgeon Peruvian Navy.

During a residence of more than ten years in South America, the greater part of which time has been spent in the military and naval service of Peru, where dysentery forms one of the most important and fatal maladies, I have had many opportunities of observing the disease, and of seeing the almost total want of success which invariably attends the early attempts of the Uuropean surgeon to treat this "scourge of armies."

The official reports show that the British army has lost in its most important expeditions more men from dysentery than from the sword. During the late campaigns in the Crimea and on the peninsula of India, this disease has been very fatal among the troops, and the practice of the surgeons in charge attended with most unfortunate results.

During four years, I was in charge of the General Hospital here (in Calloa), and a very great number of cases of dysentery passed under my treatment. I had been taught that dysentery was inflammation, and in its management, to place my chief reliance on the "lancet" and "calomel and opium." I was doomed to be miserably disappointed, and by dearly bought experience found that bleeding did no good—it only weakened the patient, without *telling* on disease; and as to mercury, that it was applicable only to certain forms of the disease; while in others it did harm, or was unnecessary: and twelve months' later experience in the burning climate of the *Ecuador*, directly under the line, has only strengthened my opinion on this point; *bleeding* being borne with less impunity in *Guayaquil* than in the more temperate climate of Peru. No doubt dysentery is inflammation, but it is *specific* inflammation, and yet yields readily to certain *specific* remedies when properly administered.

In conversation with Dr. Rios, of Lima, a native physician of great experience, he informed me that he had learned to place his reliance on large doses of *powdered ipecacuanha* and vegetable astringents, particularly the *pomgranate* or *Granada* bark (viz.: the dried rind of the fruit), in the form of decoction, and this practice, with some modifications, I adopted in the hospital.

The results of my practical experience in this disease are, that there are *five* distinct varieties of dysentery, requiring for their care different modes of treatment:—

(1.) Dysentery dependent upon indigesta and feces retained in the bowels.

(2.) Simple *specific* dysentery, which may be subdivided into *common* and *bilious*.

(3.) Dysentery complicated with *enteritis*.

(4.) Dysentery complicated with inflammation of the *liver*, acute or chronic.

(5.) Putrid, gangrenous, or malignant dysentery.

It is of vital importance that the practitioner, when called to a case of dysentery, should make a very careful examination of the patient's abdomen, in order to ascertain which of the forms of the disease he will have to treat.

Mere tenderness over the region of the colon is almost always present more or less in simple dysentery. General tenderness of the abdomen, increased on pressure, belongs to enteric dysentery ; and if the inferior lobe of the liver be found enlarged, and this viscus is tender on pressure, the case belongs to the hepatic variety.

(1.) Dysentery dependent upon indigesta and feces retained in the intestines.

*Symptoms.*—The characteristic symptoms of this form of the disease are, griping pains in the bowels, and a constant desire to go to stool ; the evacuations being very watery, mucous, or bloody and slimy, mixed with small lumps of fecal matter, called "scybala." The patient complains of a *load* in the intestines, feeling as if a ball or orange were lodged in the lower bowels. This he endeavors to throw off by violent efforts of straining, and though he feels these to be useless, he is unable to resist them. This sensation is obviously attributable to the enormously swollen state of the mucous membrane of the colon and rectum. The tongue is furred; there is some pain in the umbilical region after colic; there is *no* fever, but the heat of the skin and thirst are augmented at night; the abdomen is *hard*, but *not* tender on pressure.

*Treatment.*—These symptoms yield to the administration simply of full doses of purgative medicines: I prefer castor oil, and the free exhibition of copious enemas of olive oil and warm water. To this class of cases the observation of Dr. George Gregory is particularly applicable when he says, "but the employment of *purgatives* constitutes the most important part of the treatment of dysentery." They must be steadily persisted in until fecal evacuations have been procured, and the sensation of load in the bowels, which leads to the efforts straining, is completely removed. *Then, not till then*, may the practitioner desist from the free use of his *cathartics*. When proper fecal evacations have been produced it will be generally proper to continue the use of aperient medicines in smaller doses (the oil of sweet almonds, in doses of two ounces every morning, is an admirable remedy in these cases), and if after that the pain and discharge of mucus continue, the pulv. ipecac. comp. in doses of ten grains at bedtime, or twice a day, is an admirable remedy, well adapted to this stage of the disease.

(2.) Simple *specific* dysentery; which may be subdivided into *common* and *bilious.*

The *symptoms* are frequent calls to stool, with pain and tenemus, sometimes nothing passing but mucus *streaked* with blood, and frequently large quantities of pure blood. The evacuations are attended by acute *cutting* pain across the bottom of the abdomen, over the *public region*, and there is tenderness on pressure over the track of the *colon;* slight fever and much thirst; the tongue is covered with a dirty fur, and in the *bilious* cases there is nausea, often vomiting, and great prostration of strength, the edges of the tongue being yellowish.

*Treatment.*—I generally administer a draught composed of half a drachm to a drachm of powdered ipecacuanha, mixed in a little syrup and water; the quantity of ipecac. being increased or diminished according to the severity of the case and the strength of the patient; it should be given fasting, or early in the morning; the patient should lie in bed quiet, and not drink anything to excite vomiting. The medicine is not given as an *emetic*, but to produce its *specific* action on the disease. By the rectum I give half of the following enema, night and morning: ℞.—Radicis ipecacuanhæ contus. ℥j; aquæ ferventis lbiss; fiat infusia, cola et adde syrupi morphiæ, ℥j.—M. Ft. enema. The ipecacuanha draught should be repeated every morning for three days. It generally produces but little vomiting, but the second or third doses causes copious bilious stools, which is the desired effect of the medicine. Under this treatment patients frequently pass very large quanties of vitiated matters from the bowels, and often all the dysenteric symptoms are at once relieved. By the third or fourth morning a full dose of castor oil should be exhibited; the quantity I give is from an ounce and a half to two ounces, mixed in the form of an emulsion; small doses of castor oil only irritate, and do more harm than good in dysentery. Should, however, the case be an urgent one from the large quantites of blood discharged by stool, and not allow time for the above treatment, I administer ten grains of ipecacuanha mixed with a grain of powdered opium, every six or eight hours, and the enema of infusion of radix ipecacuanha night and morning, till the urgent symptoms are arrested. This plan I have found to succeed much better than the acetate of lead and opium, so highly extolled by some authors in this form of the disease. Under this plan of treatment, though no blood now appears in the stools, a mucous diarrhœa, with *tenesmus*, often continues to harass the patient, and it is now that the vegetable astringents are of such infinite service. I give the Granada or pomegranate bark in the form of decoction: ℞.—Corticis granati ℥ ij; aquæ lbiss; boil to a pint and strain, and add syrup of morphia ℥ j. A wine-glassful to be given every four hours. The remaining symptoms will speedily diminish under the use of this medicine. The patient should, during the time he is under treatment, drink moderately of tepid rice water, with plenty of gum Arabic dissolved in it, and sweetened to his taste. In very severe cases, the only nourishment I allow is the white of an egg beat up with a teaspoonful of powered gum Arabic, and white sugar, mixed in a teacupful of hot rice water, to be taken three times a day. The diet should be light throughout the cure. Under this plan of treatment, if the patient has been seen at all early, he is almost invariably well in a week or ten days, and relapses are of rare occurrence when common care in the diet is observed.

(3.) Dysentery complicated with *enteritis*.

*Symptoms.*—In this form of the disease, in addition to the symptoms of simple dysentery, there is high fever; acute pain in the abdomen, increased on pressure; often this part is exquisitely tender; the patient lies on his back with his knees drawn up, and cannot bear the pressure of the bedclothes The pulse is full and frequent, but seldom hard; there is extreme thirst, and the tongue is covered with a dirty white fur; the features are anxious; the urine high coloured and scanty; and in fatal cases the fever assumes the typhoid form, and the patient sinks into a state of collapse.

*Treatment.*—Judging from the fever, fulness of the pulse, and, above all, the tenderness of the abdomen, which is frequently exquisite, general blood-letting would appear to be indispensable; and, indeed, these are the cases in which we are told to bleed freely, and leech abundantly; but such a treatment will not do in the tropical climate of South America, and we must always remember that in dysentery it is specific, and not common inflammation, we have to deal with. Indeed, so badly is bleeding borne in acute enteric here, that in one severe case, "having the fear of a lancet before my eyes," I was induced to order a dozen leaches to the abdomen; a poultice was applied to the leech-bites afterwards, and they bled freely; the next morning I found my patient in such an alarming state of collapse, that it was with difficulty he was able to bear the subsequent treatment, and made a very tedious recovery. The practice I adopt in these cases, is to place my patient in a warm bath, 74° Fahr., for twenty-five minutes, and administer ℞.—Hydrarg. chloridi mit. gr. xxx.; pulvis opii gr. iij.; confectio rosæ ℥j. Ft. massa et divide en dosis No. iij.; capiat unam 6ta quaque hora. Six hours after the last dose of calomel and opium, I give a full dose of castor oil, from an ounce and a half to two ounces, in emulsion, the abdomen being freely fomented with flannels, wrung out of warm water, and if the tenesmus is urgent—which it generally is—an injection of starch and laudanum is thrown up into the rectum. If the castor oil is vomited, it should be repeated six hours after the first dose. The effect is generally a copious discharge of bilious stools, with the immediate relief of all the urgent *enteric* symptoms. When these have subsided, and the disease has been reduced to simple dysentery, it may be treated by ten-grain doses of ipecacuanha, and a grain of opium, every six or eight hours; and, finally, when all inflammatory symptoms subsided, and mucous diarrhœa with tenesmus continues to harass the patient, the decoction of Granada or pomegranate rind, and syrup of morphia, may be given, as in simple dysentery. The mercury thus administered rarely produces ptyalism, and the patient generally makes a rapid recovery.

(4.) Dysentery, complicated with acute or chronic inflammation of the liver.

*Symptoms.*—In addition to the symptoms of simple dysentery, there is pain in the right side, increased by pressure or on a deep inspiration; sometimes sympathetic pain in the right shoulder; a yellow tinge of the conjunctiva, and high-colored urine.

*Treatment.*—These cases bear bleeding better than either of the others. The whole attention of the practitioner should be directed to the affection of the liver, regarding the dysentery as only a symptom of the deranged state of the liver and portal circulation; but I rarely use the lancet, preferring topical bleeding, by leeches over the region of the liver, and applying a cupping-glass over the bites, which has many advantages over the use of poultices; you may even regulate the quantity of blood taken with great exactness, while it does not make such a mess in the bed. The bowels should be first well cleared out by calomel and rhubarb, aa gr. x, followed by a full dose of castor oil, and then the mercury should be given in divided doses, at intervals, till slight pytalism is produced. When the inflammation of the liver has been reduced by these measures, the dysentery generally subsides; if not, you may administer ipecacuanha as in simple dysentery, but it is obvious that, until the morbid state of the liver is rectified, no permanent advantage can be derived from specific treatment.

(5.) Putrid, malignant, or gangrenous dysentery.

There is a variety of this disease called malignant or putrid, from the tendency of the intestines to pass into a state of gangrene. This form of the disease is well described by Dr. Cornel. It ravaged the island of Guadaloupe in the West Indies, in 1837.

*Symptoms.*—The evacuations of bloody mucus are very frequent, and contain patches of membrane from the mucous coat of the large intestines; they have the putrid odour of gangrene strongly marked, while the room in which the patient is, as well as his breath, is impregnated with a putrid odour. The abdomen is not very painful, but greatly distended by gases. The tongue is loaded with a blackish fur; fetid sordes accumulate about the teeth and lips. The features are changed and sharpened; there is great prostration of strength, with hiccough and a viscid cold sweat; death rapidly ensues. This is the most *terrible* form of tropical dysentery; it is very common in the interior of Peru, and even in Lima, but is hardly ever seen on the sea-coast. It is highly contagious.

*Treatment.*—The remedy which, in these cases, acts like a charm, is the extract of nux vomica combined with opium in the following formula: ℞.—Extract. nuc. vomicæ gr. iv; extract. opii gr. iij.—M. Ft. pilulas No. iv. One to be taken every three hours.

The quantity of the nux vomica should be augmented to twelve grains; that is, *three* grains for a dose, according to the circumstances of the case; mucilaginous drinks, and enemas of decoction of althea, with solution of chloride of soda, are to be administered. When the patient has passed from the state of extreme danger, his case should be treated according to the symptoms that may present themselves. I am quite ignorant of the mode in which the nux vomica acts, but its administration has saved numerous individuals affected with the worst form of dysentery from the very jaws of death.

Of a very large number of cases treated on these principles, in the hospital, the deaths did not average more than *two* per cent. But I do not include in this calculation those unfortunate cases in which, from neglect or previous bad treatment, ulceration of the colon had been allowed to take place. In such cases I have found the sulphate of copper, in doses of two grains, made into a pill with half a grain of extract of opium, and taken three times a day, a remedy which, in many cases, proved successful.

Ipecacuanha has often been extolled as a remedy of great value in dysentery, but I am not aware that its use in the *full doses* I have been in the habit of employing with such almost certain success is generally known. The pomegranate rind no doubt owes its astringent virtues to the tannin it contains. It is a singular fact that, in those countries where dysentery most prevails, both ipecacuanha and the Granada or pomegranate tree are indigenous. That the bane and antidote should spring from the same soil is one of the remarkable laws of nature.

Callao, War Steamer Ucayali, April 12, 1860.

*Am. Journal Med.*

